# Supplementary material for: Evidence supported by Mendelian randomization: impact on inflammatory factors in knee osteoarthritis
Source: Front Med (Lausanne). 2024 May 28;11:1382836. doi: 10.3389/fmed.2024.1382836 (PMC11165061; doi:10.3389/fmed.2024.1382836)
Supplement: Supplementary file 1 [file Data_Sheet_1.ZIP › data/GCST90274803_5e6/803.forest.pdf]

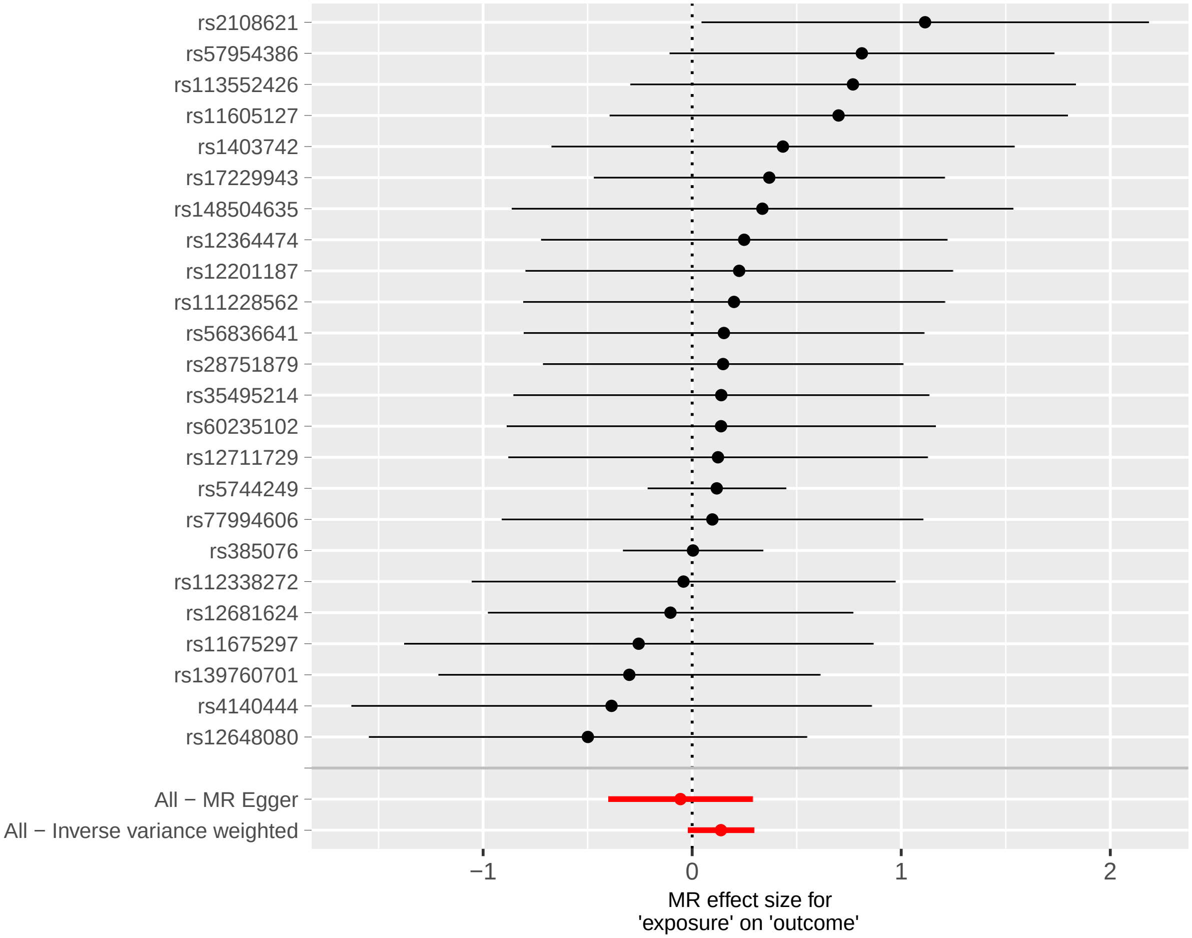
rs2108621
rs57954386
rs113552426
rs11605127
rs1403742
rs17229943
rs148504635
rs12364474
rs12201187
rs111228562
rs56836641
rs28751879
rs35495214
rs60235102
rs12711729
rs5744249
rs77994606
rs385076
rs112338272
rs12681624
rs11675297
rs139760701
rs4140444
rs12648080
All − MR Egger
All − Inverse variance weighted
−1
0
1
2
MR effect size for
'exposure' on 'outcome'
